# Synthesis of Biotin Linkers with the Activated Triple Bond Donor [*p*-(*N*-propynoylamino)toluic Acid] (PATA) for Efficient Biotinylation of Peptides and Oligonucleotides

**DOI:** 10.3390/molecules171214174

**Published:** 2012-11-30

**Authors:** Martina Jezowska, Joanna Romanowska, Burcu Bestas, Ulf Tedebark, Malgorzata Honcharenko

**Affiliations:** 1 Department of Biosciences and Nutrition, Karolinska Institutet, Novum, SE-14183 Huddinge, Sweden; E-Mail: Martina.Jezowska@ki.se (M.J.); 2 Institute of Bioorganic Chemistry, Polish Academy of Sciences, Noskowskiego 12/14, 61-704 Poznañ, Poland; E-Mail: joarom@ibch.poznan.pl (J.R.); 3 Clinical Research Center, Karolinska Institutet, Karolinska University Hospital Huddinge, Novum, SE-14186 Huddinge, Sweden; E-Mail: burcu.bestas@ki.se; 4 GE Healthcare Bio-Sciences AB, Björkgatan 30, SE-75184 Uppsala, Sweden; E-Mail: Ulf.Tedebark@ge.com

**Keywords:** biotin, streptavidin, click chemistry, biotin linker, oligonucleotide conjugates

## Abstract

Biotin is an important molecule for modern biological studies including, e.g., cellular transport. Its exclusive affinity to fluorescent streptavidin/avidin proteins allows ready and specific detection. As a consequence methods for the attachment of biotin to various biological targets are of high importance, especially when they are very selective and can also proceed in water. One useful method is Hüisgen dipolar [3+2]-cycloaddition, commonly referred to as “click chemistry”. As we reported recently, the activated triple bond donor *p*-(*N*-propynoylamino)toluic acid (PATA) gives excellent results when used for conjugations at submicromolar concentrations. Thus, we have designed and synthesized two biotin linkers, with different lengths equipped with this activated triple bond donor and we proceeded with biotinylation of oligonucleotides and C-myc peptide both in solution and on solid support with excellent yields of conversion.

## 1. Introduction

The streptavidin-biotin complex provides the basis for many important biotechnological applications and is an interesting model system for studying high-affinity protein-ligand interactions. Biotin (Btn) is a water soluble vitamin which forms extraordinary stable complexes with avidin (AVN, Mr = 67,000), neutravidin (NEVN, Mr = 60,000), a deglycosylated form of avidin, and streptavidin (STV, Mr = 66,000–75,000, isolated from the bacterium *Streptomyces avidinii*). Streptavidin is a homotetrameric 159 residue protein where each monomer of the protein binds one molecule of the vitamin biotin non-covalently with an exceptionally high affinity (*K_a_*~ 10^13^ M^−1^) [1,2]. This fact has been exploited to devise powerful and widely used tools for, e.g., visualization of protein transport (since fluorophore labeled STV is commercially available), a cell microarray [3], platform for nuclear import visualization [4] and in many other approaches [5,6,7]. 

Thus, quite a few methodologies were reported in which biotin was connected to an attachable linker. These methods rely on the synthesis of active esters [8], as well as propargyl and azide derivatives which allows use of “click chemistry” to perform conjugation [9]. The latter method (Hüisgen 1,3-dipolar cycloaddition) seems to be most attractive since: (1) it can be performed in water; (2) neither azide nor triple bond groups interfere with protecting-deprotecting strategy during synthesis of reactive parts; and (3) offers total selectivity. The strategy requires modification of the reactants into “clickable” ones by incorporating azido and triple bond donors in the respective parts. We have recently reported [10,11] a convenient method of derivatization of commercially available peptides into “clickable”-conjugates extending from either the N- or C-terminus. It is also possible to purchase azido- modified amino acids and nucleotide building blocks that allow the incorporation of this reactive group at any place in a synthetic peptide or oligonucleotide. Reported “click chemistry” procedures, that do not use an activated triple bond donor, give good yields provided that at least one reactant is used in high excess and/or in high overall concentration. From previous experiments in our own group and as well as by others, it is clear that conjugation by Cu catalyzed Hüisgen dipolar [3+2]-cycloaddition at a low concentration and using relatively large biomolecules for derivatization, like oligonucleotides or peptides, is very difficult and often virtually impossible. This is provided that the triple bond donor is not in activated form as shown in a recent reports, where comparison of pentynoic acid as triple bond donor *versus* activated PATA bond donor is discussed [10,11]. In the mentioned study the triple bond donor PATA is an activated linker which can be attached to various biomolecules like peptides and oligonucleotides and allows conjugation using 1 eq. Cu(I) (as CuSO_4_/ascorbate catalyzed or CuI catalyzed cycloaddition) at a low concentration and with excellent yields. For various studies specially for labeling of proteins it seems valuable to have access to biotin linkers which are ready for cycloadditions at a low concentration, *i.e.*, equipped with the activated triple bond donor PATA [12,13,14].

## 2. Results and Discussion

**Scheme 1:**
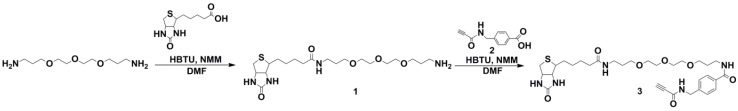
Synthesis of biotinylated linker **3** carrying the activated triple bond donor PATA.

**Scheme 2:**
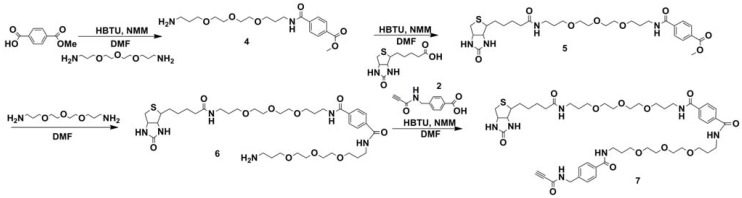
Synthesis of the longer biotinylated linker **7** carrying the activated triple bond donor PATA.

We decided to synthesize two linkers, equipped with the PATA moiety, and with different lengths (compounds **3** and **7**, Scheme 1 and Scheme 2). For labeling of some larger biopolymers like oligonucleotides a part of the molecule itself can serve as spacer. The streptavidin recognition pocket is relatively deep and biomolecules equipped with biotin typically require a spacer. A longer linker than **1** may be needed for certain purposes, which is why the longer PATA functionalized biotinylated linker **7** was also synthesized. 

### 2.1. Synthesis of Biotin Linkers Containing (p-(N-propynoylamino)toluic Acid) PATA

**Figure 1:**
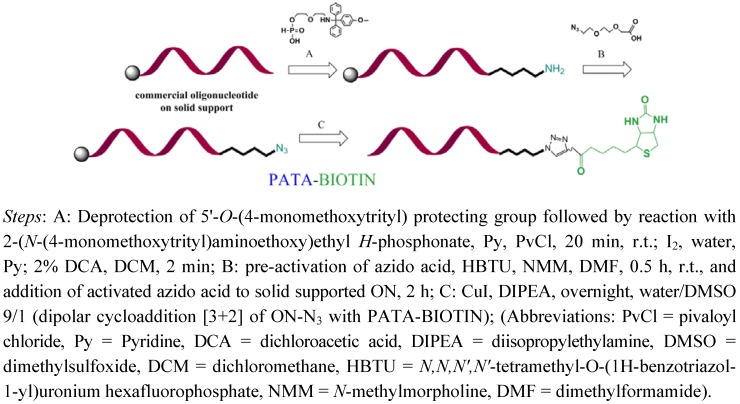
Schematic representation of biotinylation procedure for oligonucleotides on solid support.

Synthesis of the activated triple bond donor PATA (**2**), necessary for synthesis **3** and **7**, was performed as described earlier [10]. Synthesis of the shorter biotin linker **3** (Figure 1) was initiated by conjugation of activated (by HBTU/NMM see Supporting Information for details) commercial biotin with 4,7,3-trioxa-1,13-tridecanediamine. After completion of the reaction the product **1** was purified by standard column chromatography on silica (due to a lack UV absorption, MS analysis of eluted fractions was carried out). Synthesis of compound **3** was commenced by activation of the triple bond donor PATA (**2**) with HBTU/NMM followed by conjugation with compound **1** which served as an amine in this reaction. Due to the low UV absorption of the product (**3**) together with a need for high purity of the final linker we decided to use RP-HPLC (C-18 column and triethylammonium acetate buffers, pH 6.5, see Supporting Information for details) for the purification. 

It is worth to notice that using a buffer containing triethyl amine requires low temperature treatment. Heating, even slight, of activated triple bond donor PATA in the presence of triethylamine (for example for concentration purposes) may result in the formation of a side product which most likely is a triethylamine adduct (M^+^ + Et_3_NH = 732.98 for compound **3** and M^+^ + Et_3_NH = 1,082.34 for compound **7**, 10–15%) formed through Michael addition. Although not using heating during HPLC purification helps to decrease the amount of adduct formed, to avoid it completely we used “buffers” containing just acetonitrile and water (see experimental section for details).

Successful completion of the synthesis of the “short” biotinylated triple bond donor (**3**) encouraged us to synthesize a “long” biotinylated PATA linker (**7**) in a similar manner. Lack of the possibilities to detect reaction products using UV absorption can lead to some problems even though staining with for example ninhydrin or iodine can be used. To simplify purification procedures we introduced an internal linker having a UV active chromophore (terephthalate) which allowed for more ready detection of the conjugation products during TLC analysis.

The sequence of reactions which allowed us to synthesize **7** via compound **4** started from activation of monomethyl terephthalate in the same manner as for biotin (HBTU/NMM see Supporting Information for details) followed by conjugation with commercial 4,7,3-trioxa-1,13-tridecanediamine. After completion of the reaction the product **4** was purified using silica gel column chromatography. In the subsequent step activation of biotin (as described for synthesis of compound **3**) followed by conjugation with compound **4 **resulted in the desired product **5**. Since the product of that reaction is a methyl ester, aminolysis was the done with 4,7,3-trioxa-1,13-tridecanediamine (by simply dissolving compound **5** in excess of amine and leaving the reaction at 44 °C, overnight). This led to the expected non-symmetrical product **6** with one free amine. To obtain the final product the triple bond donor PATA (**2**) was activated by with HBTU and reacted with compound **6**. Reaction was completed in 3 h and the product was pre-purified by silica gel column chromatography. To obtain a high purity of the final product we used RP-HPLC in a similar manner as for the short linker, avoiding heating, to obtain the purified “long” biotinylated linker **7**. 

### 2.2. Biotinylation of C-myc Peptide and Oligonucleotides Using Biotin Linkers Containing (p-(N-propynoylamino)toluic Acid) PATA as Activated Triple Bond Donor

Successful synthesis of biotinylated linkers **3** and **7** was followed by their conjugation to oligonucleotides and C-myc peptide. As oligonucleotides we used commercial 18 and 14-mers (fully 2'-*O*-methylated in a case of 18-mer and 2'-*O*-methylated plus two LNA A building blocks used for improved hybridization properties of the 14-mer (Table 1)). Additionally cell penetrating peptide azide: azido- (C-myc) was used to achieve peptide-biotin conjugate synthesis. 

**Table 1:** Substrates and conditions of the biotinylation reaction for linkers **3** and **7**.

Substrate	Conditions	Biotin Linker	Product MS calculated/found
18merRNA_(2'-OMe)_ oligonucleotide	Solid phase	3	6796/6798
5'-CCUCUUACCUCAGUUACA-3'			
14merRNA_(2'-OMe/LNA)_ oligonucleotide	Solid phase	7	6031/6032
5'-AAAUGU ***A***ACUG***A***GG-3'			
Azido- (C-myc)	Solution phase	7	2111/2112
N_3_-L-GAAKRVKLD ^a^			

L = linker see Scheme 2; for the structure and synthesis of peptide-azide see Supplementary Information; ^a^ LNA-A building block.

**Figure 2:**
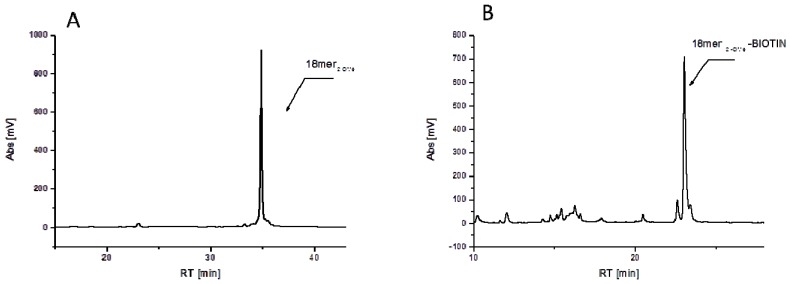
Crude chromatograms depicting: (**A**) Crude 18mer (2'-OMe) oligonucleotide and (**B**) Crude reaction mixture of the synthesis of 18mer (2'-OMe)-BIOTIN conjugate; both reaction after deprotection cleavage from the support.

Labeling with biotin is an effective, step-wise procedure, where both oligonucleotides were commercially synthesized (thus no need for an synthesizer) and easily converted into their azido-counterparts. Oligonucleotides and C-myc peptide were all purchased still attached to the solid support and subjected to reactions which transformed these molecules into “clickable ones” by addition of an azide linker (Figure 1 and Figure 2). In a case of oligonucleotides biotinylation was performed on oligonucleotide still attached to the solid support (with the conversion 55–75%, see Table 1) whereas biotinylation of C-myc peptide was done in solution.

Biotinylation of oligonucleotides relies on stepwise derivatization of commercial oligonucleotides and proceed as follows: (1) amino-linker is attached to the 5'-hydroxyl position of oligonucleotide; (2) after removal of MMTr group, terminal amino group is conjugated with 2-(2-azidoethoxy)ethoxyacetic acid; (3) CuI catalyzed Hüisgen dipolar [3+2]-cycloaddition with biotin linker containing activated triple bond donor PATA followed by ammonia treatment leads with high conversion to the desired conjugate as it is seen on the crude chromatogram depicted in Figure 2. Reaction on solid support allows for multistep reactions where excess of reagents can be removed after each step and activated triple bond donor permit for effective conversion in small scale and with minimal excess of biotin linker (usually 0.1 μmol solid supported oligonucleotide and 4 eq. of linker were used). 

**Figure 3:**
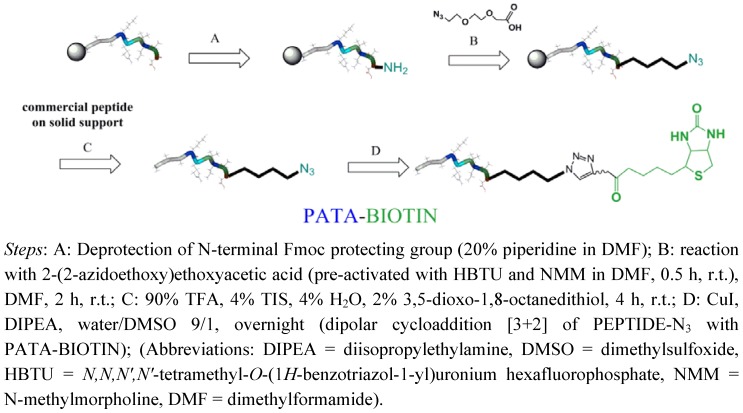
Schematic representation of biotinylation procedure for peptides.

Solution phase biotinylation of peptide was also accomplished using linker **7** in a model reaction leading to required peptide conjugate with high yields and low concentration (below 1 mM) (Figure 3 and SSupplementary Materials). Peptides with different cell penetrating properties or nuclear localization sequences can be easily purchased and converted to “clickable” ones on solid support using our recently reported method [10,11]. In short, commercial solid supported peptide is N-terminal deprotected (piperidine) and subjected to conjugation to 2-(2-azidoethoxy)ethoxyacetic acid. The reaction is followed by standard deprotection condition using TFA and HPLC purification (see SSupplementary Materials for details). Such purified peptide is easily converted to biotin conjugate using “click” reaction condition depicted in Figure 3 for PATA containing biotin linker **7**.

## 3. Experimental

### 3.1. General Materials and Methods

Acetonitrile (HPLC grade, Merck), methanol (MeOH) and dichloromethane (DCM, Fisher Scientific, Analytical Grade) were of commercial grade and used as received. Dimethylformamide (DMF) and pyridine (both from Merck and analytical grade) were additionally dried over 4A molecular sieves. Silica gel column chromatography were performed on Merck G60, TLC-analysis was carried out on precoated Silica Gel 60 F254 (Merck), with detection by UV light. NMR spectra were recorded on a Bruker AVANCE DRX-400 instrument (400.13 MHz for ^1^H, 162.00 MHz for ^31^P, 100.62 MHz for ^13^C). The oligonucleotide, 5'-ODMT-2'-OMe-CCUCUUACCUCAGUUACA [18merRNA_(2'-OMe)_], was purchased from Rasayan Inc. (Encinitas, CA, USA), and 5' ODMT- AAAUGU***A***ACUG***A***GG-3'[14merRNA_(2'-OMe/LNA)_] (Ribotask, Odense, Denmark) still attached on solid support. Solid supported peptide GAAKRVKLD (C-myc) was obtained from Eurogentech (Seraing, Belgium). The amino acid sequence was assembled by standard Fmoc synthesis on Wang resin and delivered on support with standard acid labile protecting groups: *t*-butyloxycarbonyl (Boc) for lysine, *t*-butyl ester (*O*-tBu) for aspartic acid and 2,2,4,6,7-pentamethyl-dihydrobenzofuran-5-sulfonyl (Pbf) for arginine. Nucleobase protecting groups were benzoyl for cytosine and adenine and isobutyryl for guanine. Reversed phase HPLC was carried out on a Jasco HPLC system using the following columns: Hypersil 5 μm (250 × 4.6 mm) with 1 mL/min flow rate, Kromasil 100-5-C18, 5 μm (250 × 4.6 mm) with 1 mL/min flow rate and Kromasil 100-5-C18, 5 μm (250 × 10 mm) at 4 mL/min flow rate. Buffers for reversed phase chromatography were as follows: A: H_2_O, B: H_2_O:CH_3_CN (1:1), C: 50 mM TEAA pH 6.5; D: 50 mM TEAA pH 6.5 in 50% CH_3_CN. Mass spectra (TOF-MS, ES) were obtained by using a Micromass LCT electrospray time-of-flight (ES-TOF) instrument. Molecular weights of the conjugates were reconstructed from the *m/z* values of the multiply charged ions using the mass deconvolution program (MAXENT) of the instrument, which gave values with no decimal place. *N*-methyl morpholine (NMM), biotin, 4,7,10-trioxa-1,13-tridecanediamine, were purchased from Aldrich and used without further purification.

*Biotin-(3-(2-[2-(3-aminopropoxy)ethoxy]ethoxy)propyl)amide *(**1**). The reaction flask was charged with biotin (1 eq., 1 mmol, 240 mg) and HBTU (1.05 eq., 1.05 mmol, 398 mg), evacuated on a pump and then flushed with N_2_. The solid substrates were dissolved in DMF (13 mL) and NMM (10 eq., 10 mmol, 1.1 mL) was added with a syringe. The reaction mixture was stirred for 30 min. Next 4,7,10-trioxa-1,13-tridecanediamine (2 eq., 2 mmol, 438 µL) was added and the solution was stirred for an additional 3h. After this time the reaction mixture was evaporated to dryness and the crude product was purified by column chromatography (gradient: 0–23% MeOH in DCM; due to lack of UV visibility reaction was monitored by mass spectrometry). Collected fractions containing pure product were combined and evaporated and the product was obtained as yellow oil (150 mg, 34%). ^1^H-NMR (CD_3_OD): *δ* 4.40–4.38 (1H, m), 4.22–4.19 (1H, m), 3.55–3.38 (12H, m), 3.21–3.09 (3H, m), 2.83 (1H, ½ ABq, H: *J = *4.9 Hz, *J = *14.5 Hz), 2.60 (1H, ½ ABq, H: *J = *12.7 Hz, *J = *0 Hz), −2.59 (2H, m), 2.70 (2H, t, *J* = 6.8 Hz), 2.10 (2H, t, *J* = 7.3 Hz), 1.68–1.41 (8H, m), 1.38–1.32 (2H, m). ^13^C-NMR (CDCl_3_): *δ* 27.3, 29.9, 30.2, 30.8, 33.0, 37.3, 38.2, 40.5, 41.5, 57.4, 62.1, 63.8, 70.3, 70.9, 71.60, 71.65, 71.91, 71.94. ES-MS, calc *m/z* (M-H)^+1^ 447.3, found 447.5.

*N-Biotin-(3-(2-[2-(3-aminopropoxy)ethoxy]ethoxy)propyl)amide-N’-4-(propynoylamino-methyl)-benzamide* (**3**). The reaction flask was charged with the PATA linker (**2**) (1.1 eq., 0.22 mmol, 45 mg) and HBTU (1.05 eq., 0.21 mmol, 80 mg), evacuated on a pump and then flushed with N_2_. The solid substrates were dissolved in DMF (3.5 mL) and NMM (10 eq., 2 mmol, 220 µL) was added with a syringe. The reaction mixture was stirred for 30 min. Compound **1** (1 eq., 0.2 mmol, 90 mg) was then added and the solution was stirred for an additional 2 h. After this time mass spectrometry showed completion of reaction and the mixture was evaporated to dryness. The crude product was dissolved in water and purified by RP-HPLC using a linear gradient of buffer B in A from 15%–65% in 25 min, detector at 220nm, t_R_ = 22.2 min. Concentration of pure fractions gave 64 mg (52%) of **3**. ^1^H-NMR (CDCl_3_): *δ* 7.78 (2H, d, Ar*H*, *J *= 8.0 Hz), 7.40 (2H, d, *J* = 8.0 Hz), 4.50–4.42 (3H, m), 4.30–4.18 (2H, m), 3.67–3.43 (14H, m), 3.27–3.05 (3H, m), 2.86 (1H, m), 2.72 (1H, m), 2.19–2.15 (2H, m), 1.91–1.88 (2H, m), 1.79–1.52 (6H, m), 1.46–1.36 (2H, m). ^13^C-NMR (CDCl_3_): *δ* 26.0, 28.2, 28.4, 28.8, 29.2, 35.8, 37.1, 38.8, 40.7, 43.2, 56.1, 60.0, 62.1, 69.1, 69.9, 70.3, 70.4, 70.5, 70.6, 76.7, 77.8, 126.3, 126.4, 127.4, 127.7, 133.8, 141.5, 162.5, 167.2, 173.6, 177.2. ES-MS, calc *m/z* (M−H)^+1^ 632.3, found 632.6.

*N-(3-(2-[2-(3-Amino-propoxy)ethoxy]ethoxy)propyl)terephthalamic acid methyl ester* (**4**). The reaction flask was evacuated on a pump and flushed with N_2_. Monomethyl terephthalatewas added (1 eq., 1 mmol, 180 mg) followed by HBTU (1.05 eq., 1.05 mmol, 398 mg) and DMF (30 mL). NMM (10 eq., 10 mmol, 1.1 mL) was then added with a syringe and the reaction mixture was stirred for 30 min. After that time 4,7,10-trioxa-1,13-tridecanediamine (2 eq., 2 mmol, 438 µL) was added and the solution was stirred for an additional 3 h. The reaction mixture was then evaporated to dryness. The crude product was purified by column chromatography (a linear gradient of MeOH in DCM from 0% to 20%). Fractions containing product were concentrated giving compound **4** as yellow oil (244 mg, 64%). ^1^H-NMR (CDCl_3_): *δ* 8.08 (2H, d, *J* = 8.4 Hz), 7.83 (2H, d, *J* = 8.4 Hz), 7.19 (2H, s), 7.00 (1H, m), 3.94 (3H, s), 3.79 (2H, t, *J* = 5.2 Hz), 3.68–3.49 (12H, m), 3.35 (2H, q, *J* = 5.5 Hz), 2.04–1.98 (2H, m), 1.89–1.81 (2H, m). ^13^C-NMR (CDCl_3_): *δ* 27.0, 30.0, 41.0 (2C), 52.8, 69.7, 69.9, 70.02, 70.03, 71.2, 127.7, 130.2. ES-MS, calc *m/z* (M−H)^+1^ 383.2, found 383.4.

*N-(3-(2-[2-(3-Aminopropoxy)ethoxy]ethoxy)propyl)-N'-biotinterephthalamic acid methyl ester* (**5**). To the reaction flask biotin (1 eq., 0.315 mmol, 77 mg) was added followed by HBTU (1.05 eq., 0.33 mmol, 125 mg) and the flask was evacuated on a pump and flushed with N_2_. The solid substrates were dissolved in DMF (5 mL) and NMM (10 eq., 3.15 mmol, 346 µL) was added with a syringe. The reaction mixture was stirred for 30 min. **4** (2 eq., 0.63 mmol, 240 mg) dissolved in DMF (10 mL) was then added and the solution was stirred for an additional 3 h. After completion of the reaction the mixture was concentrated and the crude product was purified by column chromatography (gradient: 0%–9% MeOH in DCM). Collected fractions were concentrated and the product was obtained as white solid. (175 mg, 91%). ^1^H-NMR (CDCl_3_): *δ* 8.08 (2H, d, *J* = 8.3 Hz), 7.93 (2H, d, *J* = 8.3 Hz), 7.44–7.43 (1H, m), 4.57–4.55 (1H, m), 4.38–4.37 (1H, m), 3.95 (3H, s), 3.68–3.59 (10H, m), 3.53–3.50 (4H, m), 3.53–3.33 (2H, m), 3.18–3.16 (1H, m), 2.84 (2H, ABq, H_1_: *J = *4.9 Hz, *J = *14.8 Hz; H_2_: *J = *13.0 Hz, *J = *0 Hz), 2.29 (2H, t, *J* = 7.3 Hz), 1.95–1.92 (2H, m), 1.78–1.67 (6H, m), 1.47–1.43 (2H, m). ^13^C-NMR (100 MHz, CDCl_3_): *δ* 26.0, 28.1, 29.2, 36.0, 38.5, 39.0, 42.0, 52.8, 55.7, 61.5, 62.5, 70.3 (2C), 70.5, 70.8, 71.0 (4C), 127.6 (2C), 130.1 (2C), 133.0, 137.0, 163.0, 166.9 (2C), 174.0. ES-MS, calc *m/z* (M−H)^+1^ 609.3, found 609.1.

*N-(3-(2-[2-(3-Amino-propoxy)-ethoxy]-ethoxy)-propyl)-N'-(3-[biotin]-propoxy)-ethoxy)-ethoxy]-propyl)-terephthalamide* (**6**). **5** (1 eq., 0.24 mmol, 150 mg) was dissolved in 4,7,10-trioxa-1,13-tridecanediamine (3.8 eq., 0.91 mmol, 200 µL) and the reaction mixture was left to stir overnight. The next day MS and TLC analysis confirmed completion of reaction and the crude product was purified by column chromatography (gradient: 0%–23% MeOH in DCM + 0.5% Et_3_N). Collected fractions were evaporated and the product was obtained as yellow oil (92 mg, 47%) ^1^H-NMR (400 MHz, CDCl_3_): *δ *7.93 (2H, d, *J* = 8.3 Hz), 7.83 (2H, d, *J* = 8.3 Hz), 4.41–4.38 (1H, m), 4.24–4.18 (1H, m), 3.60–3.33 (28H, m), 3.20–3.18 (2H, m), 3.04–3.01 (3H, m), 2.74 (2H, ABq, H_1_: *J = *4.0 Hz, *J = *12.0 Hz; H_2_: *J = *13.0 Hz, *J = *0 Hz), 2.12–2.08 (2H, m), 1.88–1.81 (6H, m), 1.68–1.41 (8H, m), 1.38–1.31 (4H, m). ^13^C-NMR (100 MHz, CDCl_3_): *δ* 24.6, 25.7, 27.0, 27.7, 27.85, 27.91, 36.2, 36.3, 37.0, 37.6, 38.3, 39.5 (2C), 54.6, 58.9, 60.8, 68.59, 68.67, 68.78 (2C), 68.85, 69.0, 69.1 (2C), 69.29 (2C), 69.30 (2C), 126.2 (4C), 126.5 (2C), 136.0 (2C), 165.9, 172.5. ES-MS, calc *m/z* (M−H)^+1^ 797.5, found 797.0.

*N-(3-[2-(2-(Biotin)-pentanoylamino]-propoxy}-ethoxy)-ethoxy]-propyl)-N'-(3-[2-(2-{3-[4-(propynoyl-aminomethyl)benzoylamino]propoxy)ethoxy)ethoxy]propyl)terephthalamide* (**7**). The reaction flask was charged with PATA (**2**) (1 eq., 0.027 mmol, 5 mg) and HBTU (1.05 eq., 0.028 mmol, 11 mg), evacuated on a pump and then flushed with N_2_. The solid substrates were dissolved in DMF (1 mL) and NMM (10 eq., 0.27 mmol, 29 µL) was added with a syringe. The reaction mixture was allowed to stir for 30 min. **6** (2 eq, 0.053 mmol, 42 mg) was then added and the solution was stirred for an additional 3 h. After this time TLC analysis showed completion of reaction and the mixture was concentrated to dryness. The crude product was purified first by column chromatography (gradient: 0%–50% MeOH in DCM +0.5% Et_3_N) and then dissolved in water and purified by RP-HPLC using a linear gradient of buffer B in A from 15%–100% in 40 min, detector at 220nm, t_R_ = 25.6. Collected fractions gave 8mg (14%) of **7**. ^1^H-NMR (CD_3_OD): *δ* 7.78 (4H, s), 7.67 (2H, d, *J* = 8.0 Hz), 7.26 (2H, d, *J* = 8.0 Hz), 4.71–4.33 (2H, m), 4.38–4.34 (2H, m), 4.20–4.17 (1H, m), 3.54–3.33 (30H, m), 3.12–3.07 (3H, m), 2.71 (2H, ABq, H_1_: *J* = 4.2 Hz, *J* = 12.3 Hz; H_2_: *J* = 12.7 Hz, *J* = 0 Hz), 2.08 (2H, t, *J* = 7.3 Hz), 1.84–1.73 (6H, m), 1.67–1.43 (H, m), 1.37–1.27 (2H, m). ^13^C-NMR (CDCl_3_): *δ* 27.3, 29.9, 30.2, 30.8 (3C), 37.3, 38.2, 39.2 (3C), 41.5, 44.3, 57.4, 62.0, 63.8, 62.0, 63.8, 70.3 (2C), 70.65 (2C), 70.73 (2C), 71.63 (2C), 71.67 (2C), 71.9 (2C), 128.90 (8C), 128.99, 129.1 (2C), 129.3, 135.3, 138.9, 143.4, 166.5, 169.5, 176.4. ES-MS, calc *m/z* (M−H)^−^ 980.5, found 980.9.

### 3.2. General Method for Biotinylation of Oligonucleotides on Solid Support

The reaction with solid supported oligonucleotides was carried out in the Eppendorf tube with screw cap. Each reagent, unless stated differently, was added to the tube with a syringe, vortexed, centrifuged and the solution was carefully removed from the solid support using a syringe. The washing steps following each reaction were carried out in the similar manner.

#### 3.2.1. Synthesis of 18-mer-(2′-OMe)-Biotin Conjugate

The commercially available solid-supported oligonucleotide (2.5 mg, 0.1 µmol CPG supported 18-mer RNA (2'-OMe)), placed in a sealed Eppendorf was treated with 2% dichloroacetic acid (DCA) solution in DCM (2 × 1 mL for 1 min) and subsequently washed with DCM (4 × 1 mL) and MeCN (2 × 1 mL). The protected MMT-aminolinker *H*-phosphonate was dissolved in anhydrous pyridine (60mM coupling solution, used 16.3 mg, 0.03 mmol in 0.5 mL pyridine) and added to the supported oligonucleotide followed by pivaloyl chloride (PvCl) in acetonitrile (1.1 eq. towards amino linker, 50 µL form stock solution: 808 µL PvCl in 10 mL MeCN) and the reaction mixture was shaken on vortex for 20 min. After this time, the coupling solution was removed and the crude product on solid support was oxidized with I_2_ (20 mg) in 1 mL pyridine/water (98:2) on vortex at RT over 15 min. The support was then washed extensively with pyridine/water (9:1) (5 × 1 mL), MeCN (5 × 1 mL), DCM (5 × 1 mL). The MMT group protecting the *H*-phosphonate amonolinker was then removed by treatment with 2%DCA in DCM (2 × 1 mL for 2 min) and the support was washed with DCM (5 × 1 mL), MeCN (2 × 1 mL) and DMF (2 × 1 mL).

In a separate Eppendorf tube, 2-(2-azidoethoxy)ethoxyacetic acid (4.7 mg, 245 eq.) was dissolved in anhydrous DMF (280 µL) and mixed with HBTU (9 mg, 240 eq.). NMM (55 µL, 5,000 µmol) was added and the mixture was shaken on a vortex for 30 min at RT. This preactivation solution was then transferred to the Eppendorf containing oligonucleotide on support and left to react at RT on vortex for 2 h. After this time, the resulting solid supported azide-modified oligonucleotide was washed with DMF (3 × 1 mL) and tBuOH/water (1:1) (2 × 1 mL). In new Eppendorf tube 4 eq. of **3** (0.25 mg) were dissolved in 70 µL of tBuOH/water (1:1) and added into the reaction vial followed by 1 eq. of diisopropyl ethyl amine (DIPEA) in 15 µL of water (from a stock solution of 2.3 µL/mL; 1 eq., 0.034 µL) and 1 eq. of CuI in 15 µL of DMSO (from stock solution of 10.1 mg CuI in 1 mL DMSO). The Eppendorf was then sealed, placed of on vortex and left for reacting at RT overnight. Next day after removing the solution from the support, the resin was washed with tBuOH/water (1:1) (3 × 1 mL), 0.05 M ethylenediaminetetraacetic acid (EDTA) disodium salt, dihydrate (2 × 1 mL) and DCM (2 × 1 mL). Next the 0.5 mL of MeOH/NH_3_ was added and the reaction mixture was stirred on a vortex at RT overnight. The reaction vial was centrifuged and the solution containing crude product was taken up carefully from the solid support and concentrated. The purification was done by RP-HPLC using a linear gradient of buffer D in C from 0% to 80% for 40 min, detection at 254 nm and oven temperature set at 50 °C. *t*_R_ = 22.9, ES-MS, calcd (M) 6796, found 6798.

#### 3.2.2. Synthesis of 14-mer-(2'-OMe)-Biotin Conjugate

The commercially available solid-supported oligonucleotide (2.5 mg, 0.1 µmol CPG supported), placed in sealed Eppendorf was treated with 2% dichloroacetic acid (DCA) solution in DCM (2 × 1 mL for 1 min) and subsequently washed with DCM (4 × 1 mL) and MeCN (2 × 1 mL). The protected MMT-aminolinker *H*-phosphonate was dissolved in anhydrous pyridine (60mM coupling solution, used 16.3 mg, 0.03 mmol in 0.5 mL pyridine) and added to the supported oligonucleotide followed by pivaloyl chloride (PvCl) in acetonitrile (1.1 eq. towards amino linker, 50 µL form stock solution: 808 µL PvCl in 10 mL MeCN) and the reaction mixture was shaken on vortex for 20 min. After this time, the coupling solution was removed and the crude product on solid support was oxidized with I_2_ (20 mg) in 1 mL pyridine/water (98:2) on vortex at RT over 15 min. The support was then washed extensively with pyridine/water (9:1) (5 × 1 mL), MeCN (5 × 1 mL), DCM (5 × 1 mL). The MMT group protecting the *H*-phosphonate aminolinker was then removed by treatment with 2%DCA in DCM (2 × 1 mL for 2 min) and the support was washed with DCM (5 × 1 mL), MeCN (2 × 1 mL) and DMF (2 × 1 mL).

In a separate Eppendorf tube, 2-(2-azidoethoxy)ethoxyacetic acid (4.7 mg, 245 eq.) was dissolved in anhydrous DMF (280 µL) and mixed with HBTU (9 mg, 240 eq.). NMM (55 µL, 5,000 µmol) was added and the mixture was shaken on a vortex for 30 min at RT. This preactivation solution was then transferred to the Eppendorf containing oligonucleotide on support and left to react at RT on vortex for 2 h. After this time, the resulting solid supported azide-modified oligonucleotide was washed with DMF (3 × 1 mL) and tBuOH/water (1:1) (2 × 1 mL). In new Eppendorf tube 4 eq. of **7** (0.39 mg) were dissolved in 70 µL of tBuOH/water (1:1) and added into the reaction vial followed by 1 eq. of diisopropyl ethyl amine (DIPEA) in 15 µL of water (from a stock solution of 1.2 µL/mL; 1 eq., 0.017 µL) and 1 eq. of CuI in 15 µL of DMSO (from stock solution of 5 mg CuI in 1 mL DMSO). The Eppendorf was then sealed, placed of on vortex and left for reacting at RT overnight. Next day after removing the solution from the support, the resin was washed with tBuOH/water (1:1) (3 × 1 mL), 0.05 M ethylenediaminetetraacetic acid (EDTA) disodium salt, dihydrate (2 × 1 mL) and DCM (2 × 1 mL). Next the 0.5 mL of ammonia was added and the reaction mixture was placed in the oven at 55 °C for overnight. Next day the solution containing crude product concentrated and purified by RP-HPLC using a linear gradient of buffer D in C from 0% to 80% for 40 min, detection at 254 nm and oven temperature set at 50 °C. *t*_R_ = 25.4, ES-MS, calcd (M) 6031, found 6032. 

#### 3.2.3. Synthesis of Peptide (C-myc)-Biotin Conjugate

The peptide azide was prepared according to our previous report [10] see the supporting information for the procedure. The biotinylation reaction was accomplished using following method: the peptide azide derivative (0.975 µmol, 1.1 mg) was dissolved in water (20 µL). The biotin linker **7** (2 eq., 1.95 µL) was dissolved in 70 µL tBuOH/water (1:1) and added to Eppendorf tube containing the peptide, followed by 1 eq. of diisopropyl ethyl amine (DIPEA) in 5 µL of water (from a stock solution of 33.6 µL/mL; 1 eq., 0.168 µL) and 2 eq. of CuI in 10 µL of DMSO (from stock solution of 45.6 mg/mL DMSO). The reaction was placed on vortex and stirred for 3 h at RT. The conjugate was purified by RP-HPLC using a linear gradient of buffer D in C from 0% to 80% for 40 min, detection at 220 nm and oven temperature set at 50 °C. *t*_R_ = 28.5, ES-MS, calcd (M) 2111, found 2112.

## 4. Conclusions

Use of the extraordinarily stable biotin/streptavidin complex pair in research has advanced steadily over the past decades. Development of new tools that take advantage of emerging technologies is imperative to accelerate progress in the field. Given the rapid influx of techniques in biochemistry and chemical biology that use the powerful [3+2] cycloaddition between azides and alkynes (“click chemistry”) for chemoselective conjugation, arming biotin with a new activated click tag provides a platform for developing novel more versatile technologies. This in turn can facilitate new approaches for exploring biological properties and important processes as well as therapeutic applications. We have shown that presented linkers are effective tool in achieving biotinylated both peptides and oligonucleotides and this reaction is effective on solid support as well as in solution. We are now using synthesized linkers and biotin-oligonucleotide conjugates for labeling of bioactive macromolecules in duplexes and the study of their action, *in vitro*, is under investigation.

## Supplementary Materials

Supplementary materials can be accessed at: http://www.mdpi.com/1420-3049/17/12/14174/s1.

Contains: Mass spectra of biotin linkers **3**, and **7**, mass spectra of oligonucleotide-biotin conjugates and peptide-biotin conjugate, description of synthesis of azido peptide (N_3_-C-myc); crude chromatograms of Biotin-oligonucleotide conjugates and crude chromatogram of peptide (C-myc)-biotin conjugate.

## References

[1] Freitag S., Le-Trong I., Klumb L., Stayton P.S., Stenkamp R.E. (1997). Structural studies of the streptavidin binding loop. Protein Sci..

[2] Weber P.C., Ohlendorf D.H., Wendoloski J.J., Salemme F.R. (1989). Structural origins of high-affinity biotin binding to streptavidin. Science.

[3] Iwasaki Y., Ota T. (2011). Efficient biotinylation of methacryloyl-functionalized nonadherent cells for formation of cell microarrays. Chem. Commun..

[4] Moreno P.M.D., Wenska M., Lundin K.E., Wrange O., Stroemberg R., Smith C.I.E. (2009). A synthetic snRNA m3G-CAP enhances nuclear delivery of exogenous proteins and nucleic acids. Nucleic Acids Res..

[5] Papalia G., Myszka D. (2010). Exploring minimal biotinylation conditions for biosensor analysis using capture chips. Anal. Biochem..

[6] Pavlickova P., Hug H. (2004). A streptavidin-biotin-based microarray platform for immunoassays. Methods Mol. Biol..

[7] Ko S.H., Gallatin G.M., Liddle J.A. (2012). Nanomanufacturing with DNA Origami: Factors Affecting the Kinetics and Yield of Quantum Dot Binding. Adv. Funct. Mater..

[8] Capaccio M., Gavalas V.G., Meier M.S., Anthony J.E., Bachas L.G. (2005). Coupling Biomolecules to Fullerenes through a Molecular Adapter. Bioconjug. Chem..

[9] Grajkowski A., Cieslak J., Gapeev A., Schindler C., Beaucage S.L. (2010). Convenient Synthesis of a Propargylated Cyclic (3'-5') Diguanylic Acid and Its “Click” Conjugation to a Biotinylated Azide. Bioconjug. Chem..

[10] Wenska M., Alvira M., Steunenberg P., Stenberg A., Murtola M., Stroemberg R. (2011). An activated triple bond linker enables “click” attachment of peptides to oligonucleotides on solid support. Nucleic Acids Res..

[11] Honcharenko M., Romanowska J., Alvira M., Jezowska M., Kjellgren M., Smith C.I.E., Strömberg R. (2012). Capping of oligonucleotides with “clickable” m_3_G-CAPs. RSC Adv..

[12] Elia G. (2012). Cell surface protein biotinylation for SDS-PAGE analysis. Methods Mol. Biol..

[13] Shaw J., Schmidt R., Snyder J., Wright L.K., Pichichero M., Michel L. Using biotinylation to determine the orientations of Pal in Escherichia coli. Proceedings of the 38th Northeast Regional Meeting of the American Chemical Society.

[14] Buneeva O.A., Medvedeva M.V., Kopylov A.T., Zgoda V.G., Medvedev A.E. (2012). Use of biotinylated ubiquitin for analysis of rat brain mitochondrial proteome and interactome. Int. J. Mol. Sci..

