# Supplementary material for: Synthesis of Biotin Linkers with the Activated Triple Bond Donor [p-(N-propynoylamino)toluic Acid] (PATA) for Efficient Biotinylation of Peptides and Oligonucleotides
Source: Molecules. 2012 Nov 30;17(12):14174–85. doi: 10.3390/molecules171214174 (PMC6269004; doi:10.3390/molecules171214174)
Supplement: Supplementary file 1 [file molecules-17-14174-s001.pdf]

Supplementary Materials

Figure S1. Mass spectra of biotin linkers.

A. Mass spectrum of biotin linker **3**.

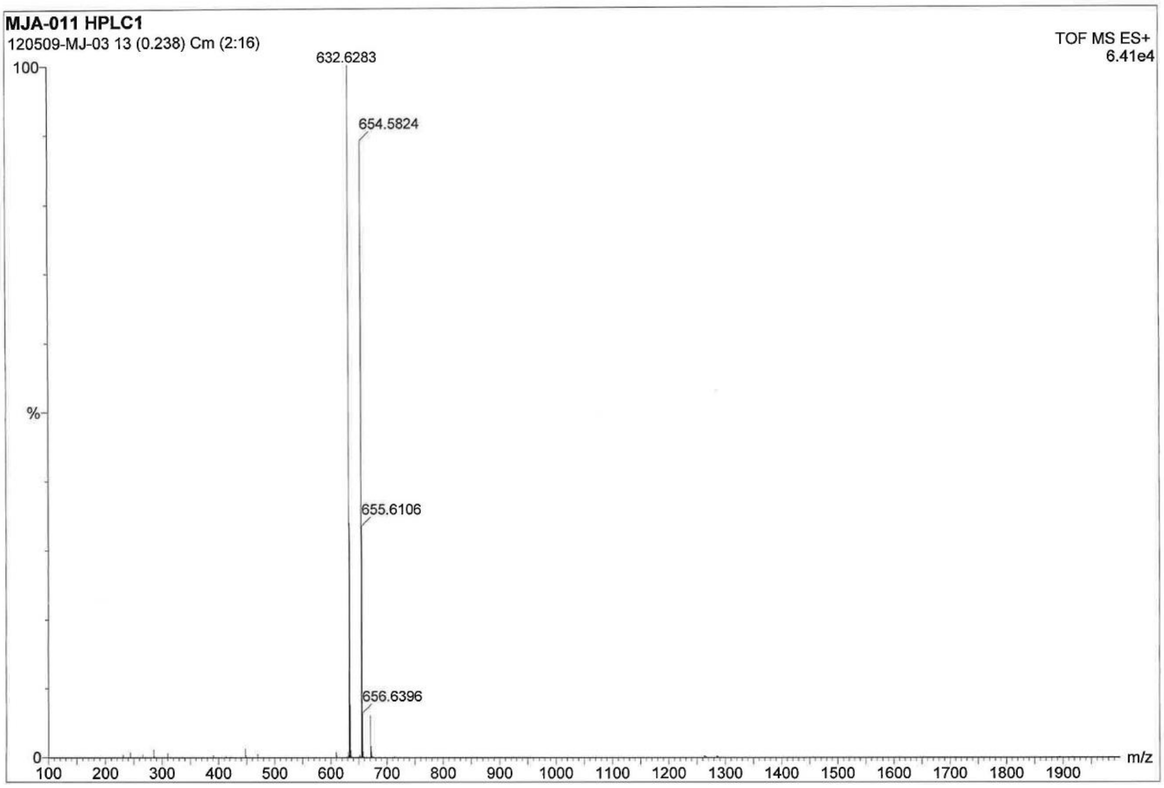

B. Mass spectrum of biotin linker **7**.

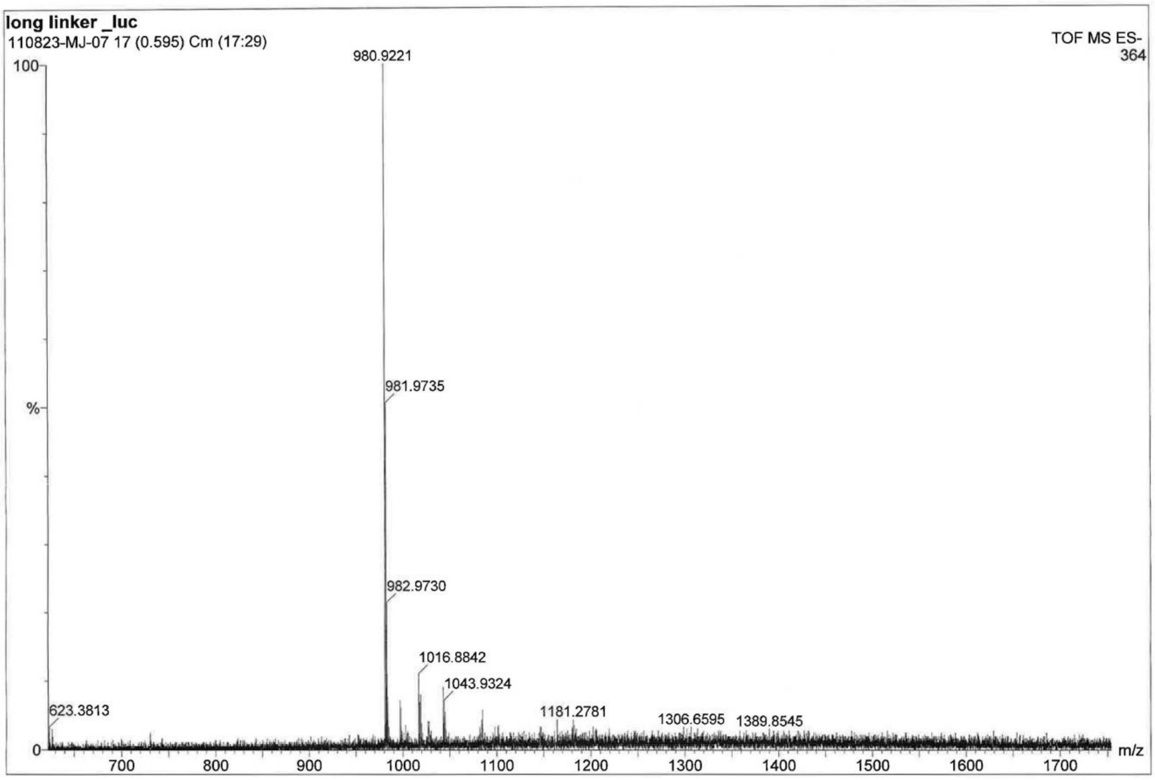

Figure S2. Mass spectra of oligonucleotide-biotin conjugate Z and Y.

A. Mass spectra of 18mer (2'-OMe)-biotin conjugate.

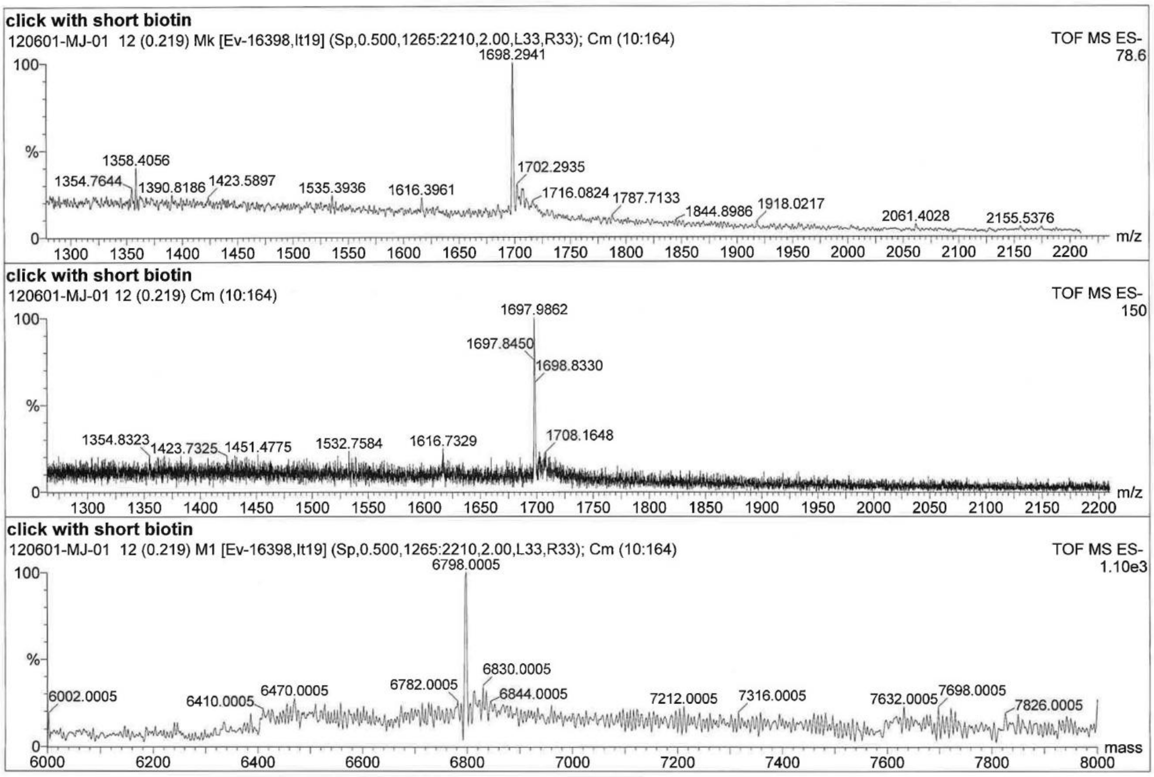

B. Mass spectra of 14mer (2'-OMe)-biotin conjugate.

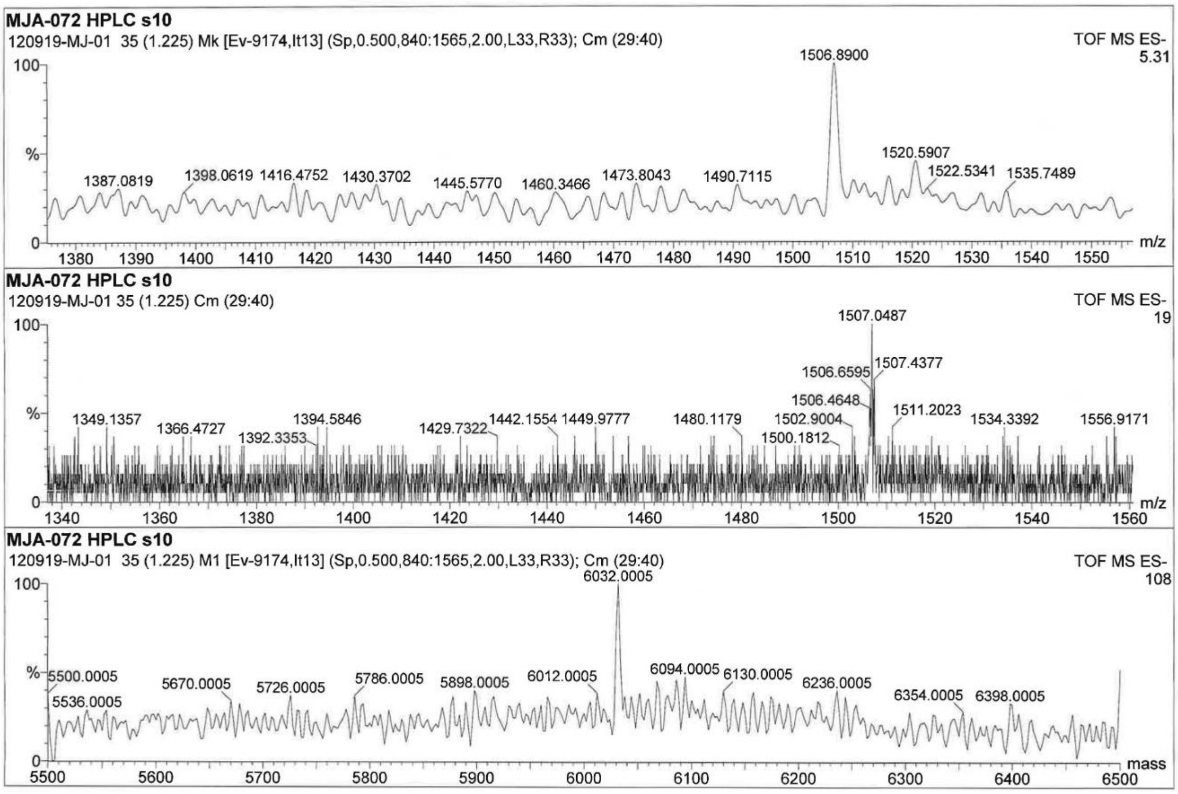

Figure S3. Mass spectra of peptide (C-myc)-biotin conjugate.

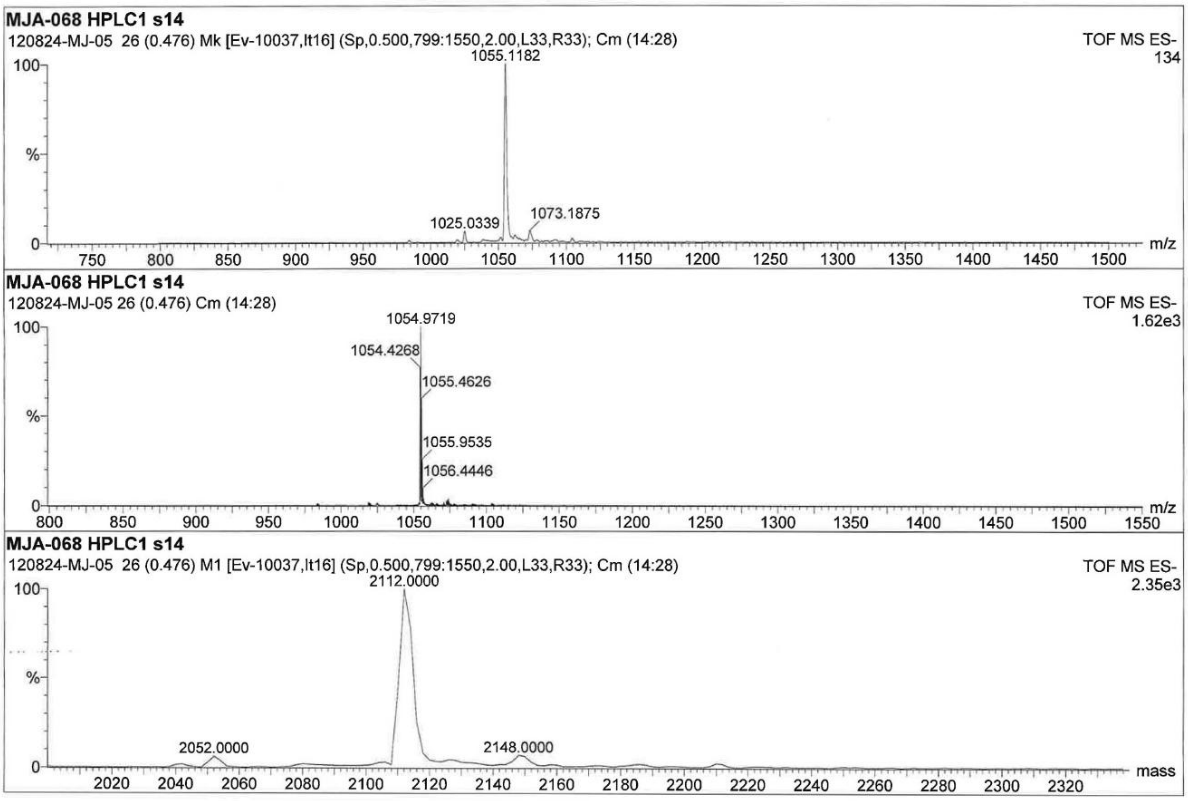

Preparation of C-myc- azide

The peptide GAAKRVKLD (0.035 mmol) still attached to solid support and with free N-terminus was placed in a sealed Eppendorf tube. In another sealed Eppendorf tube 2-(2-azidoethoxy)ethoxyacetic acid (3.3 eq.), 2-(1*H*-benzotriazole-1-yl)-1,1,3,3-tetramethyluronium hexa-fluorophosphate (HBTU, 3.3 eq.), and *N*-methylmorpholine (NMM, 6 eq.) in DMF (350 μL) were shaken for 0.5 h at r.t.. This solution was then added to the peptide on solid support. The tube was sealed and the reaction was agitated on a vortex for 2 hours at room temperature. The tube was centrifuged, the solution was carefully removed with a syringe and DMF (200 μL) was added to the support. The Eppendorf tube was then agitated on a vortex, centrifuged and the solution above the solid support was removed. This washing was repeated 5 times. After this a solution (500 μL) containing 90% trifluoroacetic acid (TFA), 4% triisopropylsilane (TIS), 4% H_2O and 2% 3,5-dioxo-1,8-octanedithiol was added and the suspension was agitated on a vortex for 4h. The tube was centrifuged and the solution was carefully collected with a syringe. Another portion of the deblocking/support cleaving solution was added and the suspension was agitated and centrifuged whereupon the solution was collected with a syringe (this washing was repeated an additional two times). The combined solutions were collected in round bottomed flask and TFA was removed by flushing air into the flask, whereupon water was added and the solution was washed with cold diethyl ether (repeated two times). The water layer was collected and concentrated *in vacuo*. Methanol was added and evaporated under reduced pressure whereupon the residue was dried under vacuum for 0.5 h giving peptide-azide (C-myc-N_3).

The peptide was dissolved in 50 mM triethylammonium acetate buffer pH 6.5 containing 15% acetonitrile and then purified by RP HPLC using a linear gradient of buffer B in A from 15–27% in 30 min, detector at 220 nm. *C-myc-azide* (7): t_R = 15.7 min, yield = 60% (HPLC); ES-MS, calc. *m/z* $(M{-}H)^{+1}$ 1128.6, found 1128.8.

Crude Chromatograms of the Synthesis of Oligonucleotide-biotin Conjugates

Figure S4. 14mer (2'-OMe)-biotin conjugate.

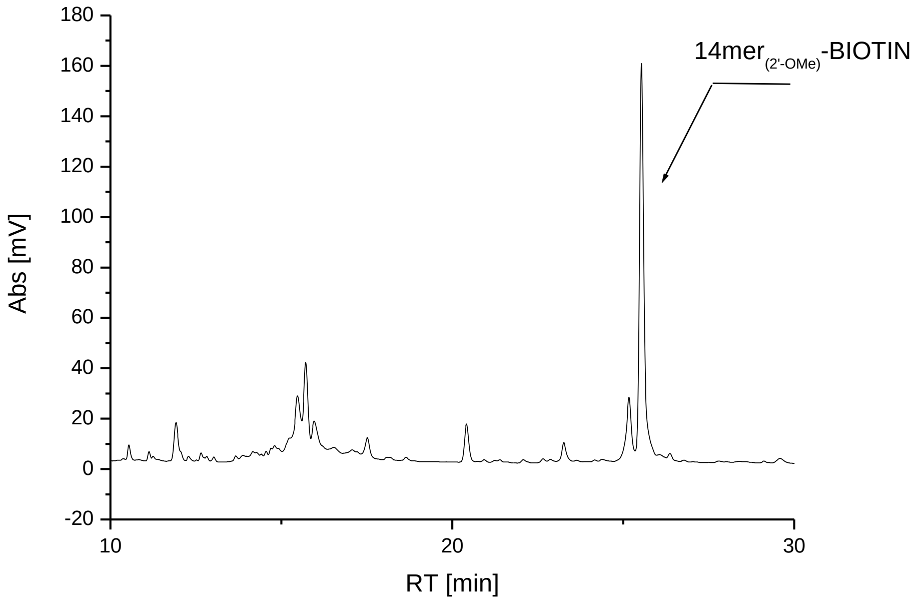

Figure S5. 18mer (2'-OMe)-biotin conjugate.

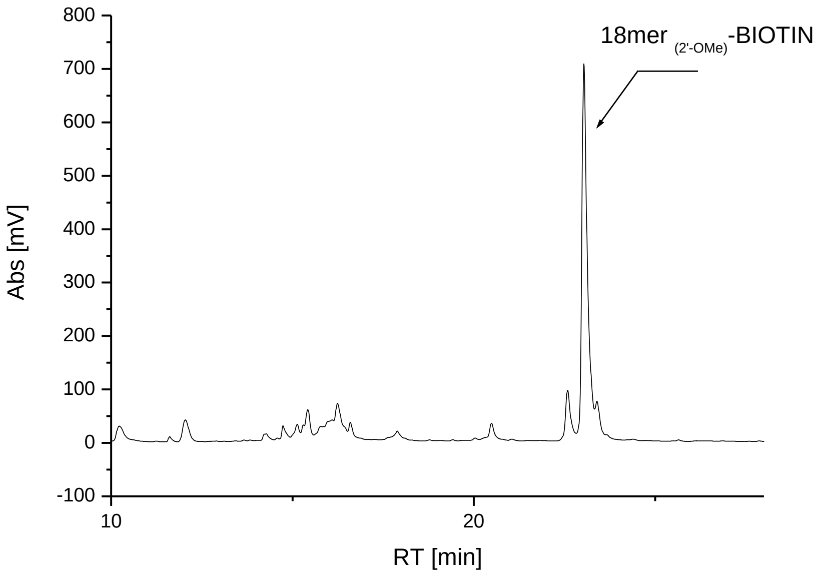

Figure S6. Peptide (C-myc)-biotin Conjugate (Crude Reaction Mixture Containing Excess of Biotin Linker).

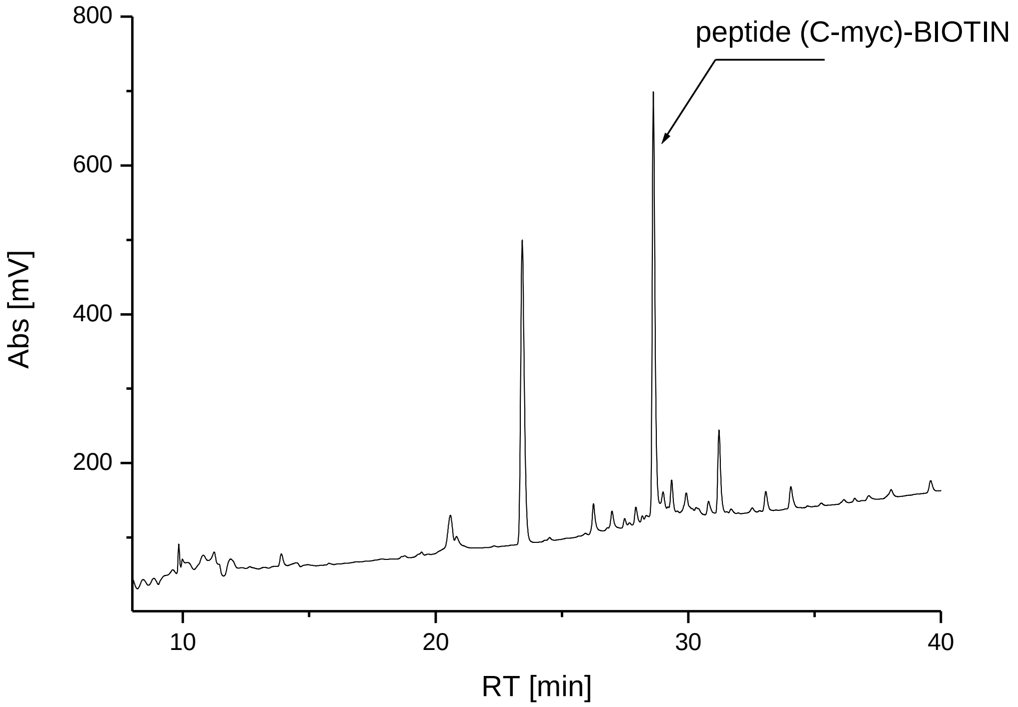

Figure S7. ^{1}H- and ^{13}C-NMR spectra of compound **3**.

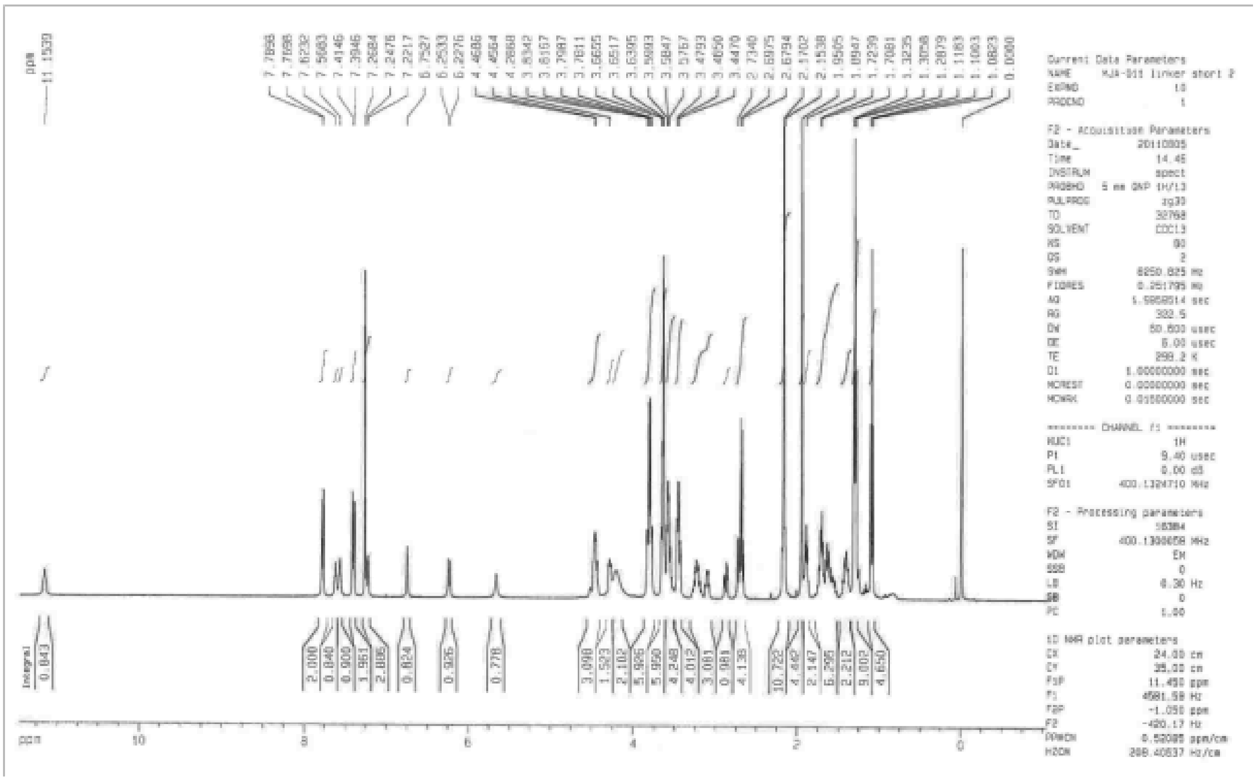

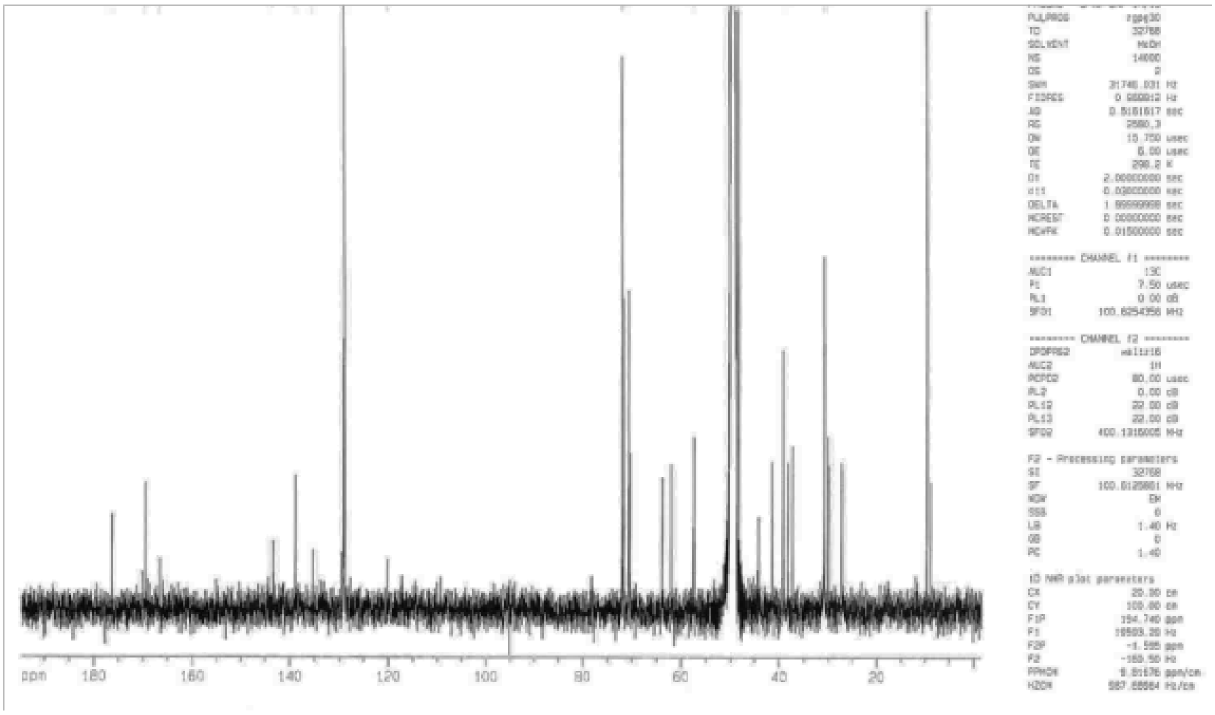

Figure S8. ^{1}H- and ^{13}C-NMR spectra of compound **7**.

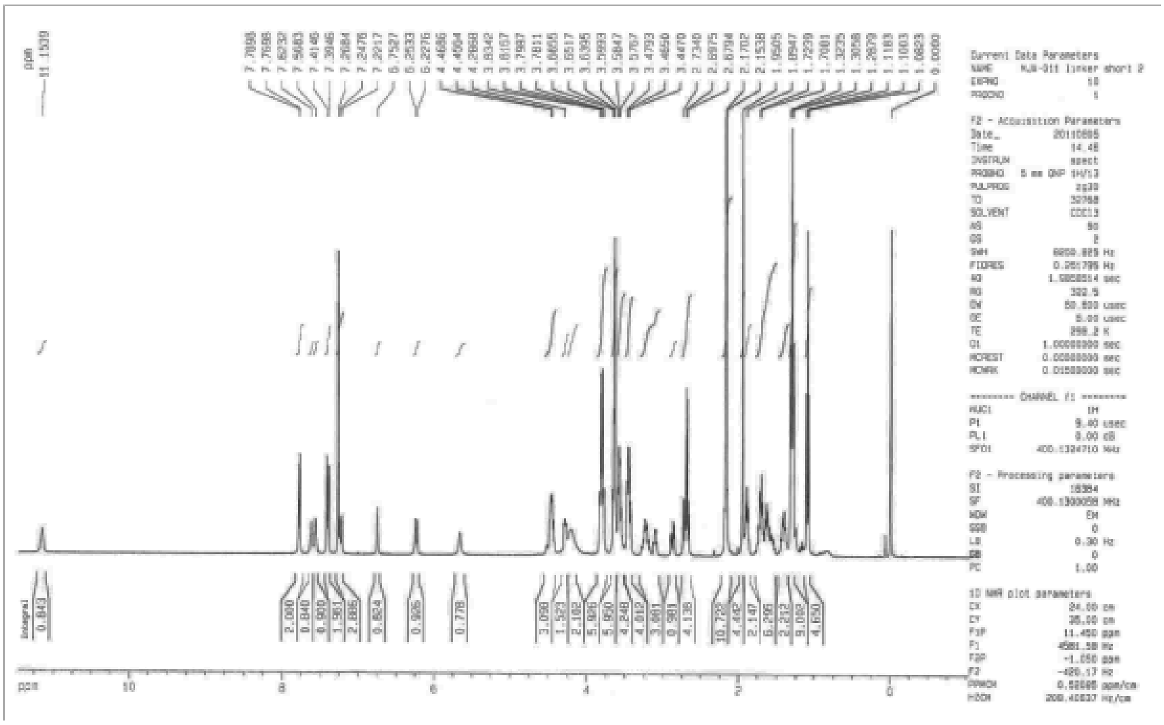

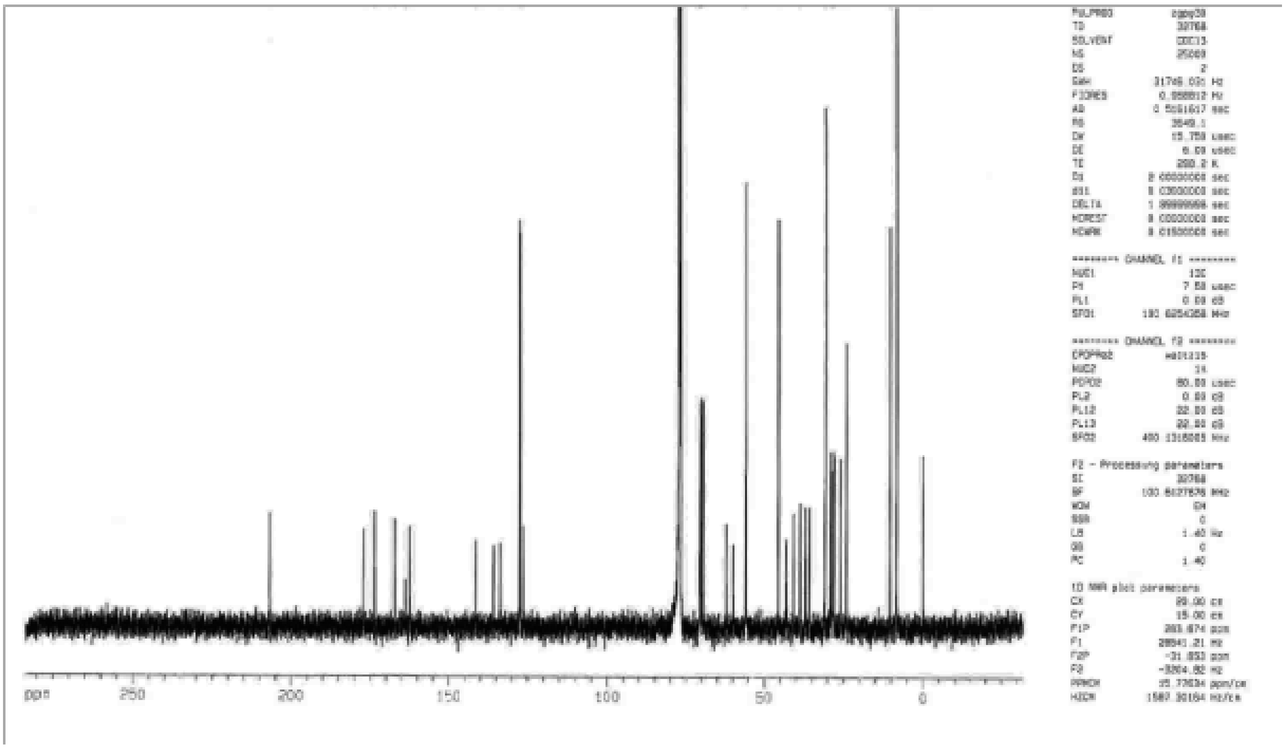
ppm
250
200
150
100
50
0
SOLVENT
CDCl3
CHANNEL f1
CHANNEL f2
F2 - Processing parameters
1D NMR plot parameters
